# Organophosphorus chemistry

**DOI:** 10.3762/bjoc.10.217

**Published:** 2014-09-04

**Authors:** Paul R Hanson

**Affiliations:** 1 Department of Chemistry, University of Kansas, 1251 Wescoe Hall Drive, Lawrence, KS 66045-7582, USA

**Keywords:** organophosphorus

Organophosphorus compounds are ubiquitous in nature, and due to their innate chemical properties, serve a fundamental role in a number of important fields. Among the more prominent features that elevate their status as a unique and versatile class of compounds, include variable oxidation states, multivalency, asymmetry and metal-binding properties. Their presence in bioactive natural products, endogenous biomolecules, small molecule therapeutic agents and pro-drugs substantiates their role in modern synthetic chemistry and chemical biology. Moreover, their central use as ligands and effectors in asymmetric catalysis, as well as key functional groups for the development of new synthetic methods, has taken the field to new heights. This Thematic Series highlights and details some of the novel methods that are advancing the field of organophosphorus chemistry.

The Thematic Series covers topics that range from new synthetic methods and phosphorus-based ligands in asymmetric catalysis to bisphosphonates as promising enzyme inhibitors. More specifically, the Thematic Series spans new methods in C–P bond formation, chiral phosphines in nucleophilic organocatalysis, chiral *N*-phosphinyl auxiliaries, cyclic phosphonamide reagents in the total synthesis of natural products, phosphinate-containing heterocycles, new routes to phosphinoyl-indoles and phosphinoyl-isocoumarins and new chemistries of H-phosphonates. The Thematic Series also details work on new metathesis-based reactions of vinyl phosphonates and phosphate tethers, novel phosphorus-based ligands in asymmetric catalysis, novel rasta resin–triphenylphosphine oxides and their use as recyclable heterogeneous reagents, the Atherton–Todd reaction, cyclic phosphonium ionic liquids with distinct properties, photo-removable phosphate protecting groups, new methods of C–H functionalization using phosphoryl-related directing groups, the exciting chemistry of substituted phosphanylidenecarbenes and phosphoryl azides, and bisphosphonate ethers as promising inhibitors of geranylgeranyl diphosphate synthase (GGDPS).

The articles and reviews capture the emerging potential of organophosphorus compounds and exciting opportunities in the field, and hopefully, will inspire and motivate investigators in the field to investigate new chemistry in this area. Taken collectively, organophosphorus chemistry embodies a broad, vibrant and continual growing scientific area with this Thematic Series highlighting recent advances in the field. We are grateful and indebted to the authors for their hard work and exciting contributions to this Thematic Series and look forward to continued contributions in this area.

Paul R. Hanson

Lawrence, July 2014

